# Return to work in younger patients with brain metastases who survived for 2 years or more

**DOI:** 10.1007/s11060-024-04840-x

**Published:** 2024-10-01

**Authors:** Carsten Nieder, Siv Gyda Aanes, Luka Stanisavljevic, Bård Mannsåker, Ellinor Christin Haukland

**Affiliations:** 1 Department of Oncology and Palliative Medicine, Nordland Hospital Trust, 8092 Bodø, Norway; 2 Department of Clinical Medicine, Faculty of Health Sciences, UiT – The Arctic University of Norway, Tromsø, Norway; 3 Department of Quality and Health Technology, Faculty of Health Sciences, SHARE – Center for Resilience in Healthcare, University of Stavanger, Stavanger, Norway

**Keywords:** Cerebral metastases, Long-term survival, Radiation therapy, Radiosurgery work ability

## Abstract

**Purpose:**

The study’s purpose was to analyze return to work and other long-term outcomes in younger patients with newly diagnosed brain metastases, treated before they reached legal retirement age, i.e. younger than 65 years.

**Methods:**

We included patients who survived greater than 2 years after their first treatment, regardless of approach (systemic therapy, neurosurgical resection, whole-brain or stereotactic radiotherapy). The primary endpoint was the proportion of patients who worked 2 years after their initial treatment for brain metastases. Outcomes beyond the 2-year cut-off were also abstracted from comprehensive electronic health records, throughout the follow-up period.

**Results:**

Of 455 patients who received active therapy for brain metastases, 62 (14%) survived for > 2 years. Twenty-eight were younger than 65 years. The actuarial median survival was 81 months and the 5-year survival rate 53%. For patients alive after 5 years, the 10-year survival rate was 54%. At diagnosis, 25% of patients (7 of 28) were permanently incapacitated for work/retired. Of the remaining 21 patients, 33% did work 2 years later. However, several of these patients went on to receive disability pension afterwards. Eventually, 19% continued working in the longer run. Younger age, absence of extracranial metastases, presence of a single brain metastasis, and Karnofsky performance status 90–100 were common features of patients who worked after 2 years.

**Conclusion:**

Long-term survival was achieved after vastly different therapeutic approaches, regarding both upfront and sequential management. Many patients required three or more lines of brain-directed treatment. Few patients continued working in the longer run.

## Introduction

Over the last decade, more and more research publications have demonstrated improved long-term survival in cancer patients with limited metastatic spread, so-called oligometastases, in settings with or without intracranial affection [1–3]. Aside from intrinsically favorable tumor biology, metastases-directed treatment such as surgical resection and stereotactic radiotherapy provides a basis for long-term survival [4, 5]. Depending on cancer type, biology features and disease dynamics, systemic treatment may also contribute to superior outcomes [6–10]. Both immune checkpoint inhibitors (ICI), antibody–drug conjugates, and targeted agents such as tyrosine kinase inhibitors (TKI) have expanded the armamentarium of efficacious options [11].

The brain metastases literature has largely focused on optimization of local control, treatment sequence and prognostic models contributing to precise identification of potential long-term survivors [12]. In addition, neurocognitive outcomes and quality of life after treatment have been evaluated [13, 14]. The latter research has promoted a management change towards a more restricted utilization of early whole-brain radiotherapy (WBRT) [15]. Little is known about survivors’ work ability, a parameter that has been studied extensively in other settings such as curative treatment of non-metastatic cancer [16–18]. Recently, our group has piloted a regional electronic health record (EHR)-based analysis of return to work in survivors of early breast cancer, which has demonstrated the feasibility of this approach [19].

The aim of the present study was to analyze return to work and other long-term outcomes in younger patients with newly diagnosed brain metastases, treated before they reached legal retirement age, i.e. younger than 65 years. In line with a recent study by Lanier et al. [20], whose endpoints were different, we included patients who survived greater than 2 years after their first treatment, regardless of approach (systemic therapy, neurosurgical resection, WBRT, stereotactic radiosurgery (SRS), stereotactic fractionated radiotherapy (SFRT)).

## Methods

For this retrospective single-institution study in a publicly-funded national healthcare system, eligible patients were identified from our previously described quality-of-care database [3, 10]. Our hospital’s clinical oncologists manage all adult cancer patients in our healthcare region (Nordland county, Norway) and administer both systemic and radiation therapy, resulting in complete data in our regional EHR. The latter also includes long-term follow-up. In order to include patients who survived greater than 2 years after their first treatment, the study was limited to the time period January 2008–December 2021. In this time period, a total of 455 adult patients received active therapy for brain metastases from solid tumors. We extracted all patients who survived greater than 2 years from the database (n = 62, 14%) and included only those treated before they reached legal retirement age, i.e. younger than 65 years (n = 28). Baseline, treatment and outcome parameters were abstracted. The primary endpoint was the proportion of patients who worked 2 years after their initial treatment for brain metastases. Outcomes beyond the 2-year cut-off were also analyzed, throughout the follow-up period. Typically, patients were evaluated every 3–4 months, including cranial and extracranial imaging studies such as brain magnetic resonance imaging (MRI). However, follow-up details were adapted to cancer type, course of disease and patient goals. Neurocognitive testing was not performed. Treatment was based on national Norwegian guidelines and discussed in primary-cancer-specific multidisciplinary tumor boards. Blood test results immediately before first brain-directed intervention were employed to retrospectively assess a validated 3-tiered prognostic model, the LabBM score [21, 22]. It includes serum hemoglobin, platelet count, albumin, C-reactive protein and lactate dehydrogenase, and is applicable to all primary cancer types. At the time of analysis in 2024, 11 patients were still in follow-up (minimum 25, maximum 128, median 77 months). Date of death was known in all other patients. Actuarial overall survival was calculated in a Kaplan–Meier analysis, utilizing IBM SPSS 29.0.1.0.

## Results

The study population included 15 women (54%) and 13 men (46%), age range 37–64 years (median 58). The most common primary cancer type was non-small cell lung cancer (NSCLC), 57% (n = 16). A majority of patients (68%, n = 19) presented with symptomatic brain metastases, while the remaining patients were diagnosed by routine imaging studies, predominantly MRI. In 15 patients (54%), brain metastases were present already at the time of the initial cancer diagnosis. The others were detected after various time intervals between 2 and 57 months. Further baseline characteristics are displayed in Table 1. It should be noticed that few patients had extracranial metastases, and that many had single brain metastasis as well as high Karnofsky performance status (KPS). Upfront and further brain-directed therapy was highly individualized. Five patients (18%) received only one brain-directed treatment. Twelve patients (43%) received three or more such treatments. The exact sequence is shown in Table 2. Twelve patients (43%) never received WBRT, but 6 of them are still alive and potentially at risk of further relapse. Only 7 patients (25%) did not receive systemic therapy at some point in time after brain metastases diagnosis.

**Table 1:** Patient characteristics (n = 28)

Parameter	n	Percent
Sex		
Female	15	54
Male	13	46
Tumor type		
NSCLC, adenocarcinoma	11	39
NSCLC, squamous cell carcinoma	3	11
NSCLC, other	2	7
SCLC	3	11
Rectal cancer	3	11
Renal clear cell cancer	3	11
Others	3	11
Number of brain metastases		
1	18	64
2–4	4	14
> 4	6	21
Extracranial metastases		
Present	7	25
Absent	21	75
LabBM score		
Favorable (0–1 points)	23	82
Intermediate (1.5–2 points)	3	11
Unknown	2	7
Upfront brain metastases treatment		
Systemic drug therapy alone	2	7
Neurosurgical resection	10	36
Whole-brain radiotherapy	7	25
Stereotactic radiotherapy	9	32
Age		
Median age, range (years)	58, 37–64	
Karnofsky performance status (KPS)		
Median KPS, range	90, 70–100	
Brain metastases size, largest lesion diameter		
Median size, range (mm)	26, 7–50	

*NSCLC* non-small cell lung cancer, *SCLC* small cell lung cancer

**Table 2:** Detailed patient characteristics, treatment and outcomes (n = 28)

Sex	Age (years)	Cancer type	Tumor PD-L1 expression	Other tumor characteristics	Diagnostic setting	Symptomatic patients’ steroid response	Interval to brain metastases (months from cancer diagnosis)	Lesion size (max., mm)	Lesion number	Karnofsky performance status	Primary tumor status	Extracranial metastases
Female	57	Adeno NSCLC, EGFR +	PD-L1 neg		Staging MR		0	13	13	90	Untreated	Bone
Female	53	Adeno NSCLC, ALK +	PD-L1 neg		Staging PET-CT		0	8	3	80	Untreated	Bone
Female	61	Squamous NSCLC	PD-L1 neg		Staging MR		0	10	1	90	Untreated	None
Male	58	Adeno NSCLC	PD-L1 high		Symptoms	Steroid improvement	23	25	1	90	Resected	None
Female	58	Adeno NSCLC	PD-L1 high		Staging PET-CT		0	18	1	90	Untreated	None
Female	52	Adeno NSCLC	PD-L1 neg		Staging CT		0	8	1	90	Untreated	None
Female	47	Adeno NSCLC, ALK +	PD-L1 low		Symptoms	Steroid improvement	0	49	1	100	Untreated	Lung
Male	44	Adeno NSCLC	Unknown		Symptoms	Steroid improvement	17	25	4	100	Controlled after chemoradiation	None
Female	61	Adeno NSCLC	PD-L1 high		Surveillance MR		12	11	1	100	Controlled after chemoradiation	None
Female	45	Breast, Her-2 +	Unknown		Symptoms	Steroid improvement	19	40	1	70	Resected	None
Female	55	Adeno NSCLC	PD-L1 high		Symptoms	Steroid improvement	6	12	7	70	Controlled after chemoradiation	None
Male	46	Melanoma	Unknown	BRAF mutation	Symptoms	Steroid improvement	0	35	2	90	Untreated	Lymph nodes
Male	58	Anaplastic NSCLC	PD-L1 high		Symptoms	Unknown	0	50	1	100	Untreated	None
Female	64	SCLC	Unknown		Symptoms	Not on steroids	12	9	9	70	Controlled after chemoradiation	Bone
Male	64	Squamous NSCLC	PD-L1 neg		Symptoms	Steroid improvement	0	28	1	90	Untreated	None
Male	57	Adeno NSCLC	Unknown		Symptoms	Steroid improvement	2	27	1	90	Resected	None
Female	37	Breast, Her-2 +	Unknown		Symptoms	Steroid improvement	17	42	6	90	Controlled after chemo/Her-2 targeted therapy	None after chemo/Her-2 targeted therapy
Male	63	Squamous NSCLC	Unknown		Staging MR		0	8	1	70	Untreated	None
Male	63	Rectal	Unknown		Symptoms	Steroid improvement	57	27	1	100	Resected	None
Male	60	Renal clear cell	Unknown		Symptoms	Steroid improvement	0	30	1	100	Untreated	Lung, adrenal
Female	64	Renal clear cell	Unknown		Symptoms	Steroid improvement	0	30	1	90	Untreated	None
Male	63	Rectal	Unknown		Symptoms	Steroid improvement	43	30	1	100	Resected	Lung, liver
Male	41	Adeno nsclc	Unknown		Symptoms	Steroid improvement	0	11	7	80	Untreated	None
Female	51	SCLC	Unknown		Staging mr		0	7	1	80	Untreated	None
Female	51	Poorly differentiated NSCLC	Unknown		Staging CT		0	29	7	90	Untreated	Lung
Male	64	Renal clear cell	Unknown		Symptoms	Steroid improvement	2	45	1	90	Resected	None
Male	57	Rectal	Unknown		Symptoms	Steroid improvement	21	29	1	80	Resected	None
Female	62	SCLC	Unknown		Symptoms	Steroid improvement	9	14	2	80	Controlled after chemoradiation	None

Sex	LabBM score	Brain metastases initial therapy	Brain metastases further therapy sequence	Any neurosurgical resection	Any SRS/SFRT	Any WBRT	Systemic therapy after brain metastases	Survival (months)	Cause of death	Work at diagnosis of brain metastases	Status at 2 years	Long-term status after > 2 years
Female	2.0	Osimertinib	None	None	None	None	Osimertinib	25 +		80% cantor	Active EC disease, on systemic 2nd line Tx, ECOG 2, preparing for disability pension claim	n/a
Female	0	Alectinib	SRS, salvage WBRT 3 Gy × 10	None	1 course	Salvage	Alectinib	39 +		Lab technician	Stable EC disease, on systemic 3rd line Tx, ECOG 1, still on sick leave	Employer-initiated layoff, reduced short-term memory
Female	2.0	SRS	Salvage WBRT 3 Gy × 10, further SRS after WBRT	None	3 courses	Salvage	After first SRS combined chemo-immunotherapy	39.5	Cerebral ischemia	80% nurse assistant	Stable EC disease, no systemic therapy, ECOG 1, still on sick leave	Disability pension, reduced short-term memory, EC disease progression
Male	1.0	Neurosurgery and post-op cavity SFRT	None	Yes	None	None	After adrenal gland metastasis combined chemo-immunotherapy	42 +		Cook	Stable EC disease on maintenance therapy, ECOG 1, still on sick leave	Extracranial progression, moved to a nursing home
Female	0	SRS	SRS	None	2 courses	None	After first SRS combined chemo-immunotherapy	51 +		Cancer-unrelated disability pension	No visible EC disease, no systemic therapy, ECOG 2	Still without further treatment
Female	Unknown	SRS	SRS	None	3 courses	None	Adjuvant chemotherapy after thoracic surgery	72 +		Selfemployed (foot care)	No visible EC disease, no systemic therapy, ECOG 1, disability pension	Still without further treatment
Female	0	Neurosurgery and post-op WBRT 2 Gy × 15	SRS, salvage WBRT 2.5 Gy × 12	Yes	3 courses	Post-op and later salvage re-irradiation	Postoperative 2 lines of TKI, later also chemo-immunotherapy	81.4	Brain metastases	Industry worker	Stable EC disease on systemic 2nd line Tx, ECOG 0, part-time back to work	Disability pension, reduced short-term memory, epilepsy, IC disease progression
Male	0	SRS	SRS	None	4 courses	None	No systemic therapy	47.9	EC disease	Office work	No visible EC disease, no systemic therapy, ECOG 0, back to work	Unexpected death, autopsy revealed active EC disease
Female	1.0	SRS	SRS, neurosurgery	Yes	2 courses	None	After thoracic disease progression combined chemoimmunotherapy	77 +		Cancer-related disability pension before brain metastases	No visible EC disease, no systemic therapy, ECOG 0	On systemic therapy for thoracic disease
Female	0	Neurosurgery and systemic therapy	Salvage WBRT 2.5 Gy × 15, later spinal RT	Yes	None	Salvage	Several lines of chemotherapy and Her-2 targeting drugs	48.3	Brain metastases	Office work	No visible EC disease, on maintenance trastuzumab for brain metastases, ECOG 1, still on sick leave	Disability pension, brain and meningeal progression
Female	0	WBRT 3 Gy × 10	SRS	None	2 courses	Upfront	Consolidation immunotherapy	78 +		Cancer-unrelated disability pension	No visible EC disease, on maintenance immunotherapy, ECOG 1	Still without further treatment
Male	0	Neurosurgery (1 tumor), SRS (other tumor)	SRS	Yes	3 courses	None	BRAF/MEK inhibitor and Ipilimumab/Nivolumab	80 +		Trucker	Stable EC disease on maintenance therapy, ECOG 1, still on sick leave	Disability pension, on systemic therapy for EC disease
Male	0	Neurosurgery	Second surgery, wbrt 2 gy × 15	Yes	None	Post-op after 2nd surgery	Immunotherapy after WBRT	52.2	Cerebral ischemia	Taxidriver	Stable EC disease on 1st line immunotherapy, ECOG 1, still on sick leave	Disability pension, extra- and intracranial progression, focus on best supportive care
Female	2.0	WBRT 3 Gy × 10	Second WBRT 2.5 Gy × 10 after 15 months	None	None	Upfront and re-irradiation	Startet first cycle chemotherapy before WBRT	28.4	Unknown	Unempløyed, supported by family	Disability pension, nursing home care, epilepsy despite imaging response after second WBRT, focus on best supportive care	Follow-up stopped after transition to best supportive care
Male	1.0	Neurosurgery and post-op WBRT 2 Gy × 15	None	Yes	None	Post-op	Together with WBRT chemotherapy and radical thoracic RT	62.5	EC disease	Teacher, parttime	No visible EC disease, no active treatment, ECOG 0, still on parttime work	Extra- and intracranial progression after 5 years, unexpected death from infection before further treatment
Male	0	Neurosurgery and post-op WBRT 2 Gy × 15	None	Yes	None	Post-op	Startet first cycle chemotherapy before WBRT	82 +		Carpenter	No visible EC disease, no active treatment, ECOG 1, back to work but heavily reduced hours	Disability pension, relapse-free
Female	0	WBRT 3 Gy × 10	None	None	None	Upfront	Maintenance Her-2 targeted therapy	122 +		40% nurse	No visible EC disease, on Her-2 targeted treatment, ECOG 0, identical work	Still working, relapse-free
Male	0.5	SFRT	SFRT, SRS, WBRT 3 Gy × 10	None	3 courses	Salvage	Startet first cycle chemotherapy before SFRT	30.2	EC disease	Cancer-unrelated disability pension	Progressive primary after initial chemoradiation, on chemotherapy, ECOG 2	Transition to best supportive care
Male	0	SRS	Neurosurgery	Yes	1 course	None	No systemic therapy	189.5	Sudden cardiac death	Engineer	No visible EC disease, no systemic therapy, ECOG 0, continued work activity	Retired, relapse-free
Male	1.0	SRS	None	None	1 course	None	After SRS sunitinib	26.9	EC disease	Cancer-unrelated 50% disability pension	Active EC disease, on systemic 2nd line Tx, ECOG 2, disability pension	Transition to best supportive care
Female	Unknown	SRS	SRS	None	2 courses	None	No systemic therapy for 5 years	153.0	EC disease	Cancer-unrelated disability pension	No visible EC disease, no systemic therapy, ECOG 0	Extracranial progression, 2 lines of systemic therapy before transition to best supportive care
Male	0	Neurosurgery and post-op cavity SFRT	SRS	Yes	2 courses	None	Chemotherapy + bevacizumab	40.8	Unknown	Engineer	Active EC disease, on systemic 2nd line Tx, ECOG 1, disability pension	Extracranial progression, another line of systemic therapy before transition to best supportive care
Male	0	WBRT 3 Gy × 10	SRS	None	2 courses	Upfront	Chemotherapy after wbrt	29.0	EC disease	Trucker	Active EC disease, after 3 lines of systemic Tx transition to best supportive care, ECOG 2, disability pension	Extracranial progression
Female	1.0	Chemotherapy followed by consolidation WBRT 3 Gy × 10	None	None	None	Upfront	No systemic therapy after wbrt, but primary tumor resection	128 +		Assistant teacher	No visible EC disease, no systemic therapy, ECOG 1, reduced work activity	Disability pension, relapse-free, after 10 years diagnosed with dementia
Female	0	WBRT 3 Gy × 10	SFRT	None	1 course	Upfront	Chemotherapy after WBRT	47.0	Unknown	Office work	Active EC disease on 3rd line systemic Tx, ECOG 2, disability pension	Follow-up stopped after transition to best supportive care
Male	0	Neurosurgery	None	Yes	None	None	No systemic therapy	95.8	Non-Hodgkins lymphoma	Retired golf instructor	No visible EC disease, no systemic therapy, ECOG 0	Relapse-free, but treated for non-Hodgkins lymphoma
Male	0.5	Neurosurgery	Neurosurgery and post-op WBRT 2.5 Gy × 15, SRS, SRS	Yes	2 courses	Salvage	No systemic therapy	29.5	Brain metastases	Office work	No visible EC disease, no systemic therapy, but need for SRS for new brain metastases despite previous WBRT, ECOG 2, disability pension	Transition to best supportive care
Female	0	WBRT 3 Gy × 10	None	None	None	Upfront	No systemic therapy	110.5	Metastatic leiomyosarcoma	Home assistant	No visible EC disease, no systemic therapy, ECOG 0, retired	Relapse-free, but treated for metastatic leiomyosarcoma before transition to best supportive care

**Fig. 1:**
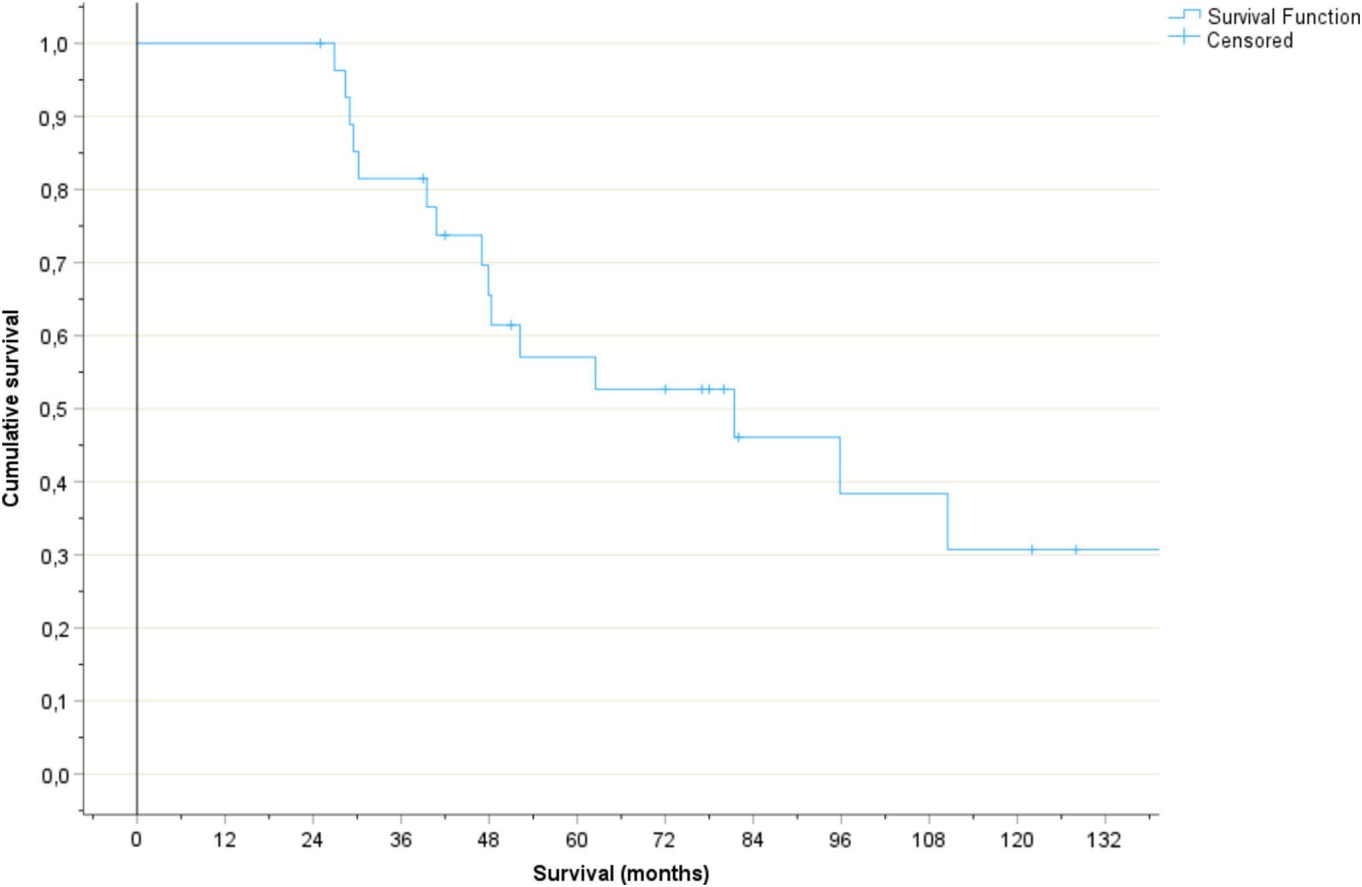
Actuarial overall survival, 11 censoring events

The actuarial median survival was 81 months and the 5-year survival rate 53% (Fig. 1). For patients alive after 5 years, the 10-year survival rate was 54%. Causes of death were uncontrolled brain metastases (n = 3), cerebral ischemia (n = 2, both treated with WBRT), uncontrolled extracranial index cancer (n = 6), second primary cancer (n = 2), sudden cardiac death (n = 1), and undocumented (n = 3, patients transferred to best supportive care in nursing homes resulting in lack of EHR data).

**Fig. 2:**
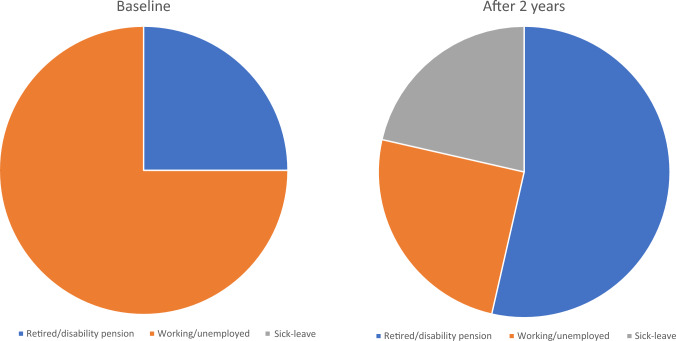
Work status at baseline and after 2 years

**Fig. 3:**
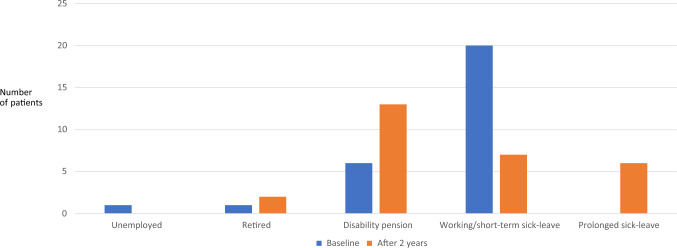
Comparison of 5 different work status categories

At brain metastases diagnosis, the following work status was documented: retired (n = 1), cancer-unrelated disability pension (n = 5), cancer-related disability pension (n = 1), unemployed (n = 1), working (n = 20, including part time work or short-term sick leave due to newly diagnosed brain metastases). At the 2-year follow-up time point, one patient was newly retired, 7 on new disability pension, and 6 on temporary sick leave. Only four had returned to the same amount of work (one managed with WBRT), while 3 worked part time (all WBRT), Figs. 2 and 3. Of 7 patients who worked after 2 years, 3 went on to receive disability pension after longer follow-up. Regarding predictive factors for return to work in these 7 patients (too few to perform statistical tests), we noticed a trend towards younger age (median 51 years), absence of extracranial metastases (6 of 7), single brain metastasis (5 of 7), and KPS 90–100 at diagnosis (6 of 7).

At 2 years, 13 patients were on active systemic therapy (46%). During follow-up, PS was recorded according to the Eastern Cooperative Oncology Group (ECOG) scale. Two-year ECOG PS was 0 in 9 patients (32%), 1 in 11 patients (39%), 2 in 7 (25%), and 3 in the remaining one (4%). During long-term follow-up, one patient developed dementia and three had physician-recorded memory deterioration (all 4 after WBRT).

## Discussion

The present study of work activity and other long-term outcomes in patients with newly diagnosed brain metastases, treated before they reached legal retirement age, i.e. younger than 65 years, provided important insights, despite its limited size and statistical power, largely precluding statistical analyses. We are not aware of larger studies providing such comprehensive work-related outcome data. The study setting was a general oncology department in a rural region, not a specialized cancer center. Therefore, referral bias is unlikely. The comprehensive regional EHR allowed for in-depth analyses of care sequence and follow-up, until the time a patient was transferred to community palliative care services, typically in a nursing home. In Norway, socio-economic consequences/financial toxicity after cancer diagnosis tend to be less severe than in many other countries [23–25].

The results can be summarized in several main categories, keeping in mind that patients older than 64 years were excluded: (1) vastly different upfront and sequential treatment strategies may lead to long-term survival, (2) many patients require three or more lines of brain-directed treatment, (3) the chance of being alive after 5 years in patients who already survived for 2 years is about 50%, (4) the chance of being alive after 10 years in patients who already survived for 5 years is about 50%, (5) typical long-term survivors are characterized by limited disease burden and good KPS, however KPS 70, presence of more than 4 brain metastases, and even extracranial metastases does not preclude survival beyond 2 years, (6) uncontrolled brain metastases can lead to death even after long time intervals from initial diagnosis, but other causes of death are much more common.

Limitations of the present study include the size of the final cohort, inadequate statistical power to develop a risk prediction model, and not having neuropsychological testing with components such as a depression questionnaire, which could add additional information about influence of mood over which patients were more likely to proceed towards work activity vs those going on disability pension.

The primary outcome of interest was work activity 2 years after brain metastases diagnosis. Based on 21 patients who worked or were temporarily unemployed or on sick-leave at diagnosis (the remaining 7 were on disability pension or retired), we found that 4 had returned to the same amount of work, while 3 worked part time (in total 7 of 21, 33%). However, 3 of these patients went on to receive disability pension after longer follow-up. Eventually, 4 of 21 patients (19%) continued working in the longer run. Even if sound statistical analyses were not feasible, it seems that younger age, absence of extracranial metastases, presence of a single brain metastasis, and KPS 90–100 are common features of patients who have returned to work after 2 years. Receiving WBRT did not preclude return to work and some of the WBRT patients reported high levels of functioning. On the other hand, several WBRT patients developed cognitive decline or even dementia and lethal cerebral ischemic events (possibly unrelated). Toxicity-mitigating strategies such as hippocampal sparing and administration of memantine were not employed [26, 27]. The same was true for longitudinal neurocognitive testing. At present, WBRT is utilized in a much more tailored and restricted fashion than in the earlier years of our study.

Two years after initial diagnosis of brain metastases, most survivors had good ECOG PS (0–1 in 71%). Many were on systemic therapy (46%), either ongoing first-line/maintenance drugs or palliative second- or third-line treatments. It is therefore difficult to provide firm conclusions about the role of different treatments in preventing return to work, especially in a retrospective manner. We did not have information about personal economy, support and other potential drivers of patients’ decision to stop their work activity, because the oncology care providers did not elaborate on such aspects in their written EHR notes. It is possible to discuss all these aspects during dedicated consultations in the context of a prospective longitudinal study. Many previous work ability studies (performed in other, typically early cancer settings) relied on questionnaires that were sent to cancer survivors, with variable return rates [18, 19, 28]. This type of study allows for in-depth analyses of socio-economic parameters and also open answers, which may shed light on the complex decision making that cancer patients and their families are facing. It is also important to notice that availability of rehabilitation and employers’ attitudes towards return to work may interfere with return to work rates [29]. The latter are generally much higher in non-brain-metastases studies than in the present analysis. Our previous study of female breast cancer survivors showed that the majority of those ≤ 65 years of age at diagnosis returned to work [19]. In a very small study of 8 long-term survivors who had received WBRT for metastatic melanoma, 6 were able to return to their previous work [30]. In contrast, in a study of 42 meningioma patients, 52% were able to return to work [31]. Of 125 patients with glioblastoma (mean age 48 years, median survival 23 months), 21 (18%) went back to work, most on a part-time basis [32]. Of the patients who were alive at 12, 18, and 24 months after diagnosis, 14%, 15%, and 28%, respectively, were working. Patients going back to work were significantly younger, had significantly fewer comorbidities, and had a significantly different distribution of socio-professional groups, with more patients belonging to higher paying/ranking categories.

A Swedish study of patients with metastatic breast cancer (mBC) with maximum age of 63 years analyzed working net days (WNDs) during the year after mBC diagnosis [33]. The study compared the time periods 1997–2002 and 2003–2011. Thirty-seven percent of patients had > 180 WNDs during the first year with mBC. Work activity was significantly higher in those younger than 51 years, and those with soft tissue, visceral or brain metastases as first metastatic site, as well as sickness absence < 90 net days in the year before mBC diagnosis, suggesting limited comorbidities. Mean (standard deviation) WNDs were 135 (140) and 161 (152) for patients diagnosed with mBC in 1997–2002 and 2003–2011, respectively (p = 0.046). Given that numerous efficacious mBC treatments were approved after 2011, a follow-up study testing the hypothesis that WNDs have increased even more would be of high relevance.

Lanier et al. performed a single-institution retrospective study of 300 patients treated with SRS from 2001 to 2019 for brain metastases, who survived for at least 2 years [20]. Actuarial median overall survival was 4.9 years and time to distant brain failure 1.5 years. Twenty-eight patients (9%) underwent subsequent WBRT. Only 101 patients (34%) never had any further brain metastases at a median follow-up time of 3.3 years. In our study, brain failure resulting in administration of several lines of brain-directed therapy was also very common. It would be interesting to generate prospective data on the contribution of these failures to work ability, also with regards to tailored interventions that aim at avoiding disability pension claims. Overall, larger studies addressing survivorship issues including but not limited to work activity are urgently needed in this era of more efficacious treatment, increasing survival and declining risk of neurologic death.

## Data Availability

All data is provided in the Tables.
